# Oxidation of Al-Co Alloys at High Temperatures

**DOI:** 10.3390/ma13143152

**Published:** 2020-07-15

**Authors:** Patrik Šulhánek, Marián Drienovský, Ivona Černičková, Libor Ďuriška, Ramūnas Skaudžius, Žaneta Gerhátová, Marián Palcut

**Affiliations:** 1 Faculty of Materials Science and Technology in Trnava, Slovak University of Technology in Bratislava, J. Bottu 24, 91724 Trnava, Slovakia; patrik.sulhanek@stuba.sk (P.Š.); marian.drienovsky@stuba.sk (M.D.); ivona.cernickova@stuba.sk (I.Č.); libor.duriska@stuba.sk (L.Ď.); zaneta.gerhatova@stuba.sk (Ž.G.); 2 Faculty of Chemistry and Geosciences, Vilnius University, Naugarduko g. 24, 01513 Vilnius, Lithuania; ramunas.skaudzius@chgf.vu.lt

**Keywords:** Al alloy, Co alloy, complex intermetallic, oxidation kinetics, oxide scale

## Abstract

In this work, the high temperature oxidation behavior of Al_71_Co_29_ and Al_76_Co_24_ alloys (concentration in at.%) is presented. The alloys were prepared by controlled arc-melting of Co and Al granules in high purity argon. The as-solidified alloys were found to consist of several different phases, including structurally complex m-Al_13_Co_4_ and Z-Al_3_Co phases. The high temperature oxidation behavior of the alloys was studied by simultaneous thermal analysis in flowing synthetic air at 773–1173 K. A protective Al_2_O_3_ scale was formed on the sample surface. A parabolic rate law was observed. The rate constants of the alloys have been found between 1.63 × 10^−14^ and 8.83 × 10^−12^ g cm^−4^ s^−1^. The experimental activation energies of oxidation are 90 and 123 kJ mol^−1^ for the Al_71_Co_29_ and Al_76_Co_24_ alloys, respectively. The oxidation mechanism of the Al-Co alloys is discussed and implications towards practical applications of these alloys at high temperatures are provided.

## 1. Introduction

Co-based superalloys are promising materials for high temperature structural applications because of their high melting points and favorable mechanical properties [1,2,3]. Applications of these alloys include gas turbines, aircraft engines, and chemical reactors [4,5,6]. The Co-based superalloys are often alloyed with chromium to provide oxidation resistance [7,8]. The superalloys alloyed with Cr form a compact chromia scale (Cr_2_O_3_) on their surface. Nevertheless, at high temperatures and high oxygen partial pressures, the Cr_2_O_3_ scale is prone to degradation. During long-term oxidation, volatile high-valent oxides of Cr, such as CrO_2_ and CrO_3_, start to form at the expense of Cr_2_O_3_. This effect is called “chromia evaporation” and is often pronounced in humid atmospheres [9]. The loss of protective chromia scale leads to a reduced life span of the Co-based superalloys. Several authors have, therefore, investigated the possibility of improving the high temperature oxidation stability of Co superalloys by alloying with Al [10,11,12]. Al-based alloys form a protective oxide scale composed of alumina (Al_2_O_3_). Al_2_O_3_ has a lower growth rate compared to Cr_2_O_3_ and is non-volatile. Furthermore, Al is a non-transition element. It has a smaller tendency to form complex oxides with transition metals compared to Cr, thereby reducing the risk of scale spallation over time. The formation of alumina scales may be achieved by pack aluminizing the alloy’s surface [13,14,15]. The application of Al-Co coatings could significantly extend the alloy’s lifetime [16,17,18,19,20,21,22].

**Figure 1:**
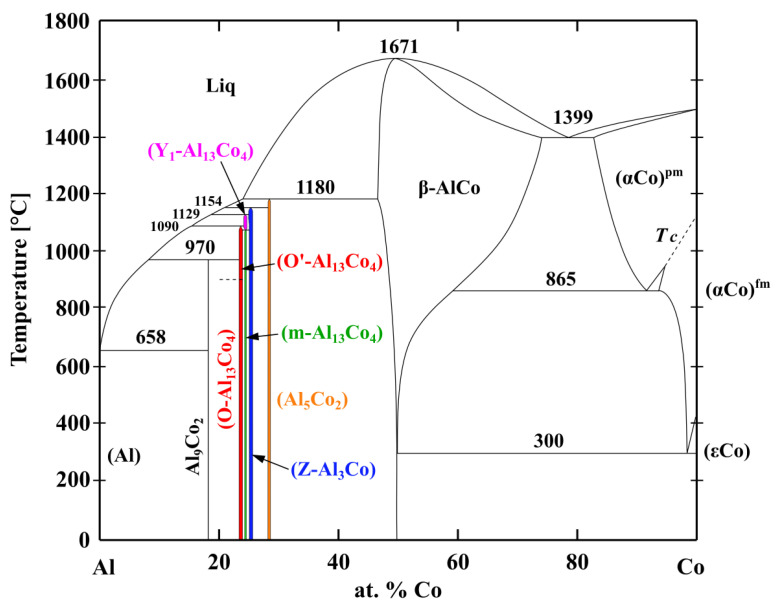
Phase diagram of the Al-Co binary system, redrawn from [23,24].

Aluminides are aluminum-based intermetallic compounds with transition metals. Cobalt aluminides are interesting for high temperature applications since they possess a combination of high melting points and good corrosion resistance. At ~18–30 at.% Co, different structurally complex aluminides in the Al-Co binary system have been observed (Figure 1, [23,24]). These include Al_9_Co_2_ (P2_1_/C), Al_5_Co_2_ (P6_3_/mmc), Z-Al_3_Co (P2/m) and family of Al_13_Co_4_ phases containing m-Al_13_Co_4_ (C2/m), O-Al_13_Co_4_ (Pmn2_1_), O’-Al_13_Co_4_ (Pnma), Y_1_-Al_13_Co_4_ (C2/m) and Y_2_-Al_13_Co_4_ (Immm) [25,26,27,28,29,30,31,32,33,34]. Although the individual cobalt aluminides are brittle [35], the Al-Co precipitates may significantly strengthen the Al alloys [36,37]. The presence of Al_5_Co_2_ and Al_13_Co_4_ intermetallic compounds (IMCs) may also be beneficial in Co-based alloys as they may greatly improve the alloy’s wear resistance [38]. Most aluminides form protective alumina scales with large resistance against corrosion [39].

High temperature corrosion studies of cobalt aluminides are limited. Metal oxidation is a heterogeneous reaction taking place in several elementary steps [40]. In the first step, a gaseous oxygen molecule is transported to the metal surface. Upon approaching the solid phase, the adsorption of oxygen molecules to the metal substrate occurs. Subsequently, the oxygen is dissociated to atoms and later reduced to the O^2−^ anions. In parallel, oxidation of metal atoms takes place at the metal-oxide interface. Recently, M. Wardé et al. studied the adsorption of oxygen on the Al_9_Co_2_ (001) and Al_13_Co_4_ (100) surfaces at high temperatures and reduced oxygen pressures [41]. At the surfaces, only Al–O bonding was observed. Al–O distances were also calculated from first principles [41,42]. The Al–O lengths were shorter in comparison with Co–O distances. The obtained Al–O distances were in agreement with the typical distances of oxygen adsorption on the Al (111) surface, as well as with the Al–O distances in Al_2_O_3_.

Al-rich Al-Co alloys belong to a relatively new group of complex metallic alloys (CMA, [43,44]). These materials contain structurally complex phases including quasicrystals. The structurally complex phases have non-periodically ordered atomic arrangements [45,46]. Consequently, the properties of CMA are different from those observed in traditional materials [43]. The quasicrystalline surfaces have a good adhesion and low coefficient of friction [47]. Owing to their high hardness and good oxidation resistance, the Al-TM alloys (TM = transition metal) are suitable for high temperature coatings [48,49,50,51]. The Al-Co CMAs are also interesting for catalytic and hydrogen generation applications [52,53,54,55]. The corrosion studies of Al-Co alloys are limited. Lekatou et al. investigated the corrosion behavior of an Al_82_Co_18_ (metal concentrations are given in at.%) alloy in saline solution [56]. Three methods of alloy preparation were investigated: casting, arc-melting and free sintering. The alloy prepared by arc-melting was found to be the most corrosion-resistant. The Al_82_Co_18_ alloy was composed of (Al), Al_9_Co_2_ and m-Al_13_Co_4_. The complex intermetallic m-Al_13_Co_4_ in the alloy was found to have the highest corrosion resistance. Recently, we have investigated the corrosion behavior of as-solidified and near equilibrium Al-Co alloys in various environments [24,57,58]. The alloys were composed of various intermetallic phases. In HCl and NaCl solutions, a pitting corrosion occurred. A higher corrosion resistance of structurally complex Z-Al_3_Co phase in Cl-containing electrolytes was observed. The difference in the corrosion behavior could be ascribed to the strong covalent character of metallic bonds in the structurally complex Al-Co phases which prevents aluminum diffusion. The studies also suggest that the existence of an electrical contact between different alloy phases play an important role in the overall alloy’s corrosion behavior.

High temperature oxidation studies of Al-Co alloys have been limited to Co-rich alloys only. Zhang et al. investigated the oxidation behavior of Co-5 at.% Al and Co-10 at.% Al alloys at 973 and 1073 K [59,60]. The oxide scales were primarily composed of cobalt oxide and cobalt-aluminum oxide. The oxides grown on these alloys were relatively thick (>10 μm after 24 h). Only a limited amount of Al_2_O_3_ was found at the inner side of the scales. Irving et al. studied the oxidation behavior of Co-xAl alloys (0 < x < 32.4 at.%) at 1073–1273 K [61]. The authors found that 20–25 at.% Al is necessary to form a continuous alumina scale. As such, larger Al concentrations are needed to improve the corrosion resistance of Al-Co alloys.

To our best knowledge, oxidation studies of Al-rich Al-Co alloys have not been reported yet. In the present work, we aim to study the oxidation behavior of Al-rich Al-Co alloys in air at 773–1173 K. Two alloys, Al_71_Co_29_ and Al_76_Co_24_ (composition in at.%) were prepared by arc-melting. The composition of the Al_71_Co_29_ alloy was chosen close to Al_5_Co_2_ (71.4 at.%). The composition of the Al_76_Co_24_ alloy was close to Al_13_Co_4_ (76.5 at.%). The high temperature oxidation of the alloys was studied with the aim to identify the role of the alloy’s chemical composition and microstructure on the overall corrosion behavior.

## 2. Materials and Methods

The alloys with nominal compositions Al_71_Co_29_ and Al_76_Co_24_ (metal concentrations in at.%) were prepared from Co and Al lumps (purity of 99.95%, smart-elements.com) by arc-melting. The melting was conducted in MAM-1 arc melter (Edmund Buehler Ltd., Bodelshausen, Germany) in high purity argon. A piece of Ti (oxygen getter) was melted first to remove residual traces of oxygen in argon. The Co and Al lumps were placed in the center of Cu mold and rapidly melted by striking an arc from tungsten cathode. The homogeneity of the molten samples was improved by repeated arc-melting. Subsequently, the melts were solidified on a water-cooled Cu mold to form button ingots. The as-solidified alloys were removed from the arc-melter, cut into smaller specimens by diamond blade, and prepared for oxidation experiments. The samples were ground with grade 1200 abrasive paper and polished with diamond suspensions down to 1 μm surface roughness.

The oxidation behavior of the polished alloys was studied in flowing synthetic air (20 vol. % O_2_ and 80% vol. % N_2_). The air flow rate was 20 mL/min. Isothermal oxidation experiments were performed at 773, 973 and 1173 K. The polished samples were placed in an alumina crucible and heated from room temperature to peak temperature by heating rate 20 K/min. The oxidation time was 30 h. The mass gain of the samples was recorded continuously in a chamber of NETZCH STA 409 CD thermogravimeter (NETZSCH-Gerätebau GmbH, Selb, Germany). After oxidation, the samples were cooled down, mounted in epoxy resin, and cut perpendicularly to the reaction interface. The cross-sections of the specimens were prepared by wet grinding and polishing and subjected to microscopy observation.

The microstructure and chemical composition of the alloys was studied by scanning electron microscopy. During experiments, a JEOL JSM-7600F microscope (JEOL Ltd., Tokyo, Japan), equipped with an energy-dispersive x-ray spectrometer X-max (EDS), was used. The EDS was operated by INCA software (Oxford Instruments Nanoanalysis, Bucks, UK). Regimes of secondary electrons (SE) and backscatter electrons (BSE) were used during imaging. The accelerating voltage of the electron beam was 20 kV and working distance was 15 mm. Furthermore, a Panalytical Empyrean PIXCel 3D diffractometer was used for the phase analysis (Malvern Panalytical Ltd., Malvern, UK). The diffractometer was working with Bragg–Brentano geometry and used CoK*_α_*_1_ radiation. The X-ray beam was generated at 40 kV and 50 mA.

## 3. Results and Discussion

### 3.1. Alloy Microstructure and Constitution before Oxidation

**Figure 2:**
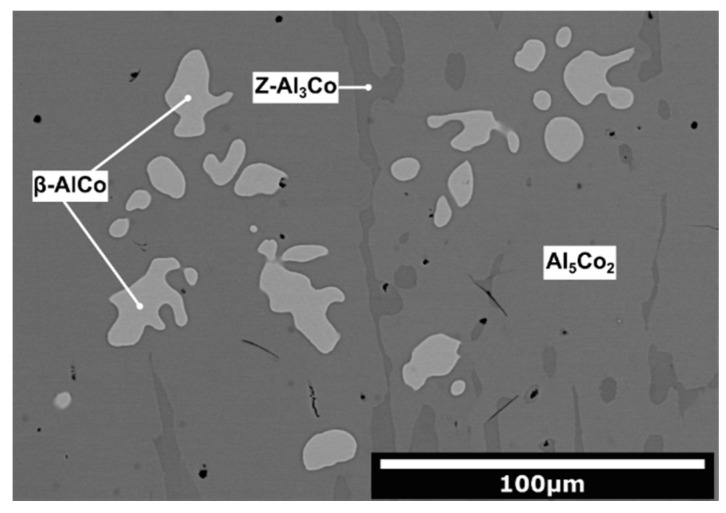
Microstructure of the as-solidified Al_71_Co_29_ alloy.

The Al_71_Co_29_ and Al_76_Co_24_ alloys were prepared by arc-melting. The microstructure of the as-solidified Al_71_Co_29_ alloy is presented in Figure 2. In this alloy, three different microstructure constituents have been found. The image was acquired in backscatter electron mode to provide element resolution. The chemical composition of the constituents, measured by EDS point analysis, is provided in Table 1. The dendritic constituents have a significantly higher Co concentration (44.8 at.%) compared to the remainder of the alloy. The other two constituents have 28.1 and 25.4 at.% Co, respectively. The black areas, found in the microstructure, are pores.

**Figure 3:**
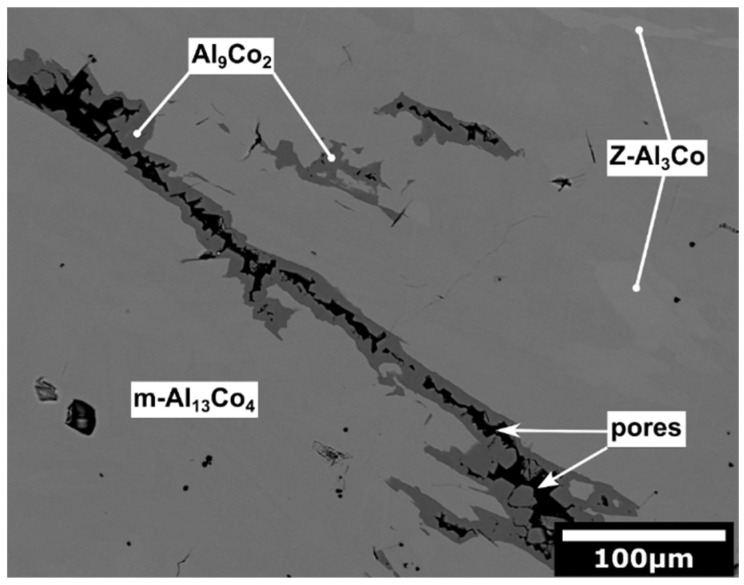
Microstructure of the as-solidified Al_76_Co_24_ alloy.

The microstructure of the as-solidified Al_76_Co_24_ alloy is presented in Figure 3 in BSE imaging mode. In this alloy, three different microstructure constituents have been found (light grey, medium grey and dark grey). The light grey constituent in the Al_76_Co_24_ alloy has 74.4 at.% Al and 25.6 at.% Co (Table 1). As such, its chemical composition is comparable to chemical compositions of the dark grey constituent observed in the as-solidified Al_71_Co_29_ alloy (Figure 2). The medium grey constituent contains 75.2 at.% Al and 24.8 at.% Co. The dark grey constituent of the as-solidified Al_76_Co_24_ alloy has 81.5 at.% Al and 18.5 at.% Co. The black areas located within the dark grey constituent are pores (Figure 3).

A phase assignment of the alloy’s microstructure constituents is presented in Table 1. The assignment has been made based on the experimental chemical composition of the constituents obtained by EDS and crystal structure of the phases identified by XRD in our previous work [57,58]. In the studied alloys, altogether five different phases have been identified: *β*-AlCo, Al_5_Co_2_, Z-Al_3_Co, m-Al_13_Co_4_ and Al_9_Co_2_ (Table 1).

The presence of different phases in the alloys indicates that non-equilibrium processes had been taking place during rapid solidification. The dendritic shape of *β*-AlCo in the as-solidified Al_71_Co_29_ alloy suggests that it solidified first, directly from the melt. The dendritic *β*-AlCo is located inside the Al_5_Co_2_ phase. Therefore, Al_5_Co_2_ was probably formed by partial transformation of *β*. The Al_5_Co_2_ phase is located next to Z-Al_3_Co (Figure 2). As such, Z-Al_3_Co was probably formed by peritectic reaction of Al_5_Co_2_ with the surrounding melt.

The as-solidified Al_76_Co_24_ alloy was found to consist of Z-Al_3_Co, m-Al_13_Co_4_ and Al_9_Co_2_, respectively (Figure 3). The m-Al_13_Co_4_ phase is located next to Z-Al_3_Co. As such, it was probably formed by peritectic reaction of Z-Al_3_Co with the melt. The Z-Al_3_Co phase has a lower Al concentration compared to m-Al_13_Co_4_ (Table 1). Al_9_Co_2_, observed in the as-solidified Al_76_Co_24_ alloy, is located next to m-Al_13_Co_4_ (Figure 3). As such, Al_9_Co_2_ was probably formed by peritectic reaction of m-Al_13_Co_4_ with the remaining melt. It was observed to be porous (Figure 3). The pores are usually formed by vacancy migration, with sub-grains and natural surfaces serving as sinks [62,63]. In the present case, the pores were found in the interior of Al_9_Co_2_. The preferential pore formation indicates that the pores could be a result of rapid transformation of the liquid Al_9_Co_2_ into solid in the final step of solidification.

### 3.2. Oxidation Behavior

**Figure 4:**
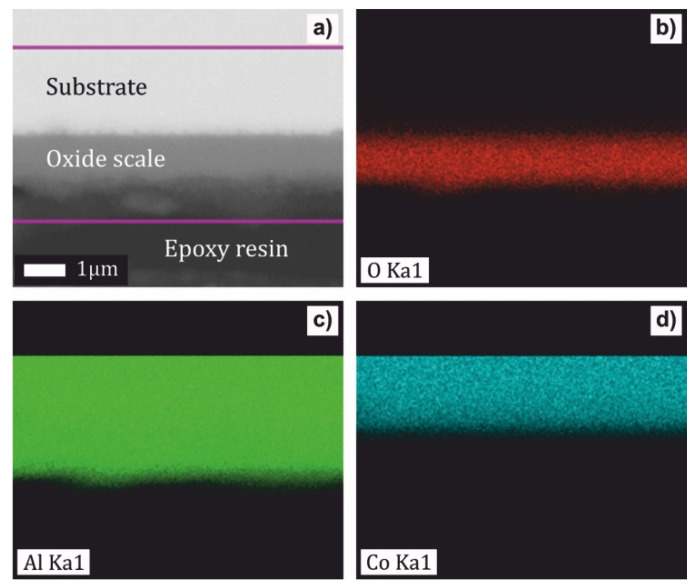
Cross section of the Al_71_Co_29_ alloy after oxidation at 1173 K for 30 h (**a**) and energy-dispersive x-ray spectrometer X-max (EDS) element maps for O (**b**), Al (**c**) and Co (**d**).

The oxidation behavior of the as-solidified Al_76_Co_24_ and Al_71_Co_29_ alloys was studied in flowing synthetic air at 773, 973 and 1173 K. The microstructure and chemical composition of the oxide scale were investigated by SEM/EDS. The cross-section of the Al_71_Co_29_ alloy after oxidation at 1173 K is presented in Figure 4. The oxide scale was homogeneous. EDS element maps are included in Figure 4b–d. The scale was found to be composed of aluminum oxide.

**Figure 5:**
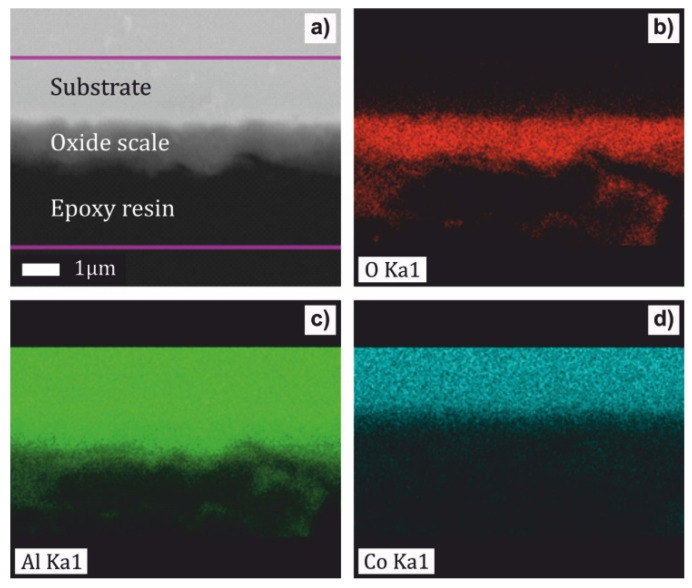
Cross section of the Al_76_Co_24_ alloy after oxidation at 1173 K for 30 h (**a**) and EDS element maps for O (**b**), Al (**c**) and Co(**d**).

The cross-section image of the Al_76_Co_24_ alloy after oxidation at 1173 K is presented in Figure 5. The thickness of the oxide scale was approximately 1 μm after 30 h of oxidation. The chemical composition of the oxide scale was studied by EDS analysis. Results presented in Figure 5b show that the scale was predominantly composed of Al_2_O_3_. A small amount of Co (~ 2 at.%) was also detected in the scale, however, this result is attributable to a possible interference of the bulk alloy signal.

**Figure 6:**
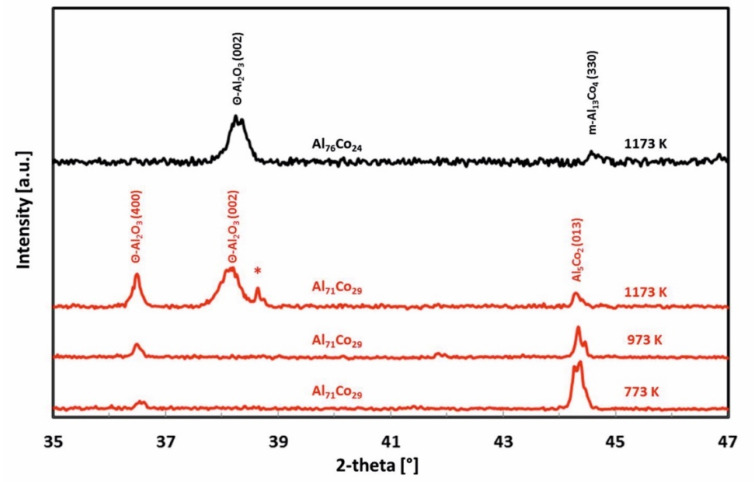
Room temperature XRD patterns of the scales formed on the Al_71_Co_29_ and Al_76_Co_24_ alloys. For the discussion of the peak marked with an asterisk (*), please refer to the article text.

The phase constitution of the oxide scale was studied by room temperature X-ray diffraction. The diffraction patterns of the Al_71_Co_29_ alloy are presented in Figure 6. In the alloy, peaks corresponding to *θ*-Al_2_O_3_ have been identified. *θ*-Al_2_O_3_ is a metastable alumina phase [64,65]. *θ*-Al_2_O_3_ structures are based on a cubic close packing of oxygen anions. The cubic close packing of oxygen anions of *θ*-Al_2_O_3_ is deformed monoclinic. The thermodynamically stable *α*-Al_2_O_3_ adopts a corundum structure. *θ*-Al_2_O_3_ is a transition phase. It transforms into stable forms of alumina during long term annealing [65]. Metastable *θ*-Al_2_O_3_ has been found in oxidized Al-Cu-Fe alloys studied previously [66]. It was formed initially with an orientational relationship to the substrate. At 1173 K, *θ*-Al_2_O_3_ was found to slowly transform into *α*-Al_2_O_3_ with an increasing oxidation time (70 h, [66]).

**Figure 7:**
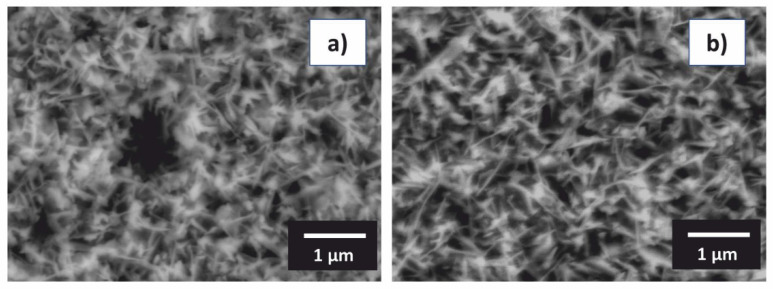
Blade-like morphology of alumina scale formed on the oxidized Al_71_Co_29_ alloy (**a**) and Al_76_Co_24_ alloy (**b**) during oxidation at 1173 K.

At 1173 K, an orientation of *θ*-Al_2_O_3_ in (002) crystallographic plane has been found (Figure 6). The same behavior was also observed for the Al_76_Co_24_ alloy (Figure 6). This observation indicates a preferential crystal growth. The morphology of alumina scale formed on the Al_71_Co_29_ and Al_76_Co_24_ alloys after oxidation at 1173 K for 30 h is given in Figure 7. The scale had a blade-like structure. The platelet-like scale morphology is indicative of rapid outwards growth. Alumina scales grow by counter-diffusion of aluminum and oxygen [67]. The ions, however, diffuse faster in polycrystalline alumina at near-atmospheric oxygen partial pressures. The scale morphology is indicative of rapid diffusion through the scale. A grain boundary diffusion was probably the preferred transport path for the Al^3+^ and O^2−^ ions in the scale.

The un-indexed peak next to *θ*-Al_2_O_3_ (002), marked with an asterisk in Figure 6, is an alumina peak. The closest match was found for hexagonal form of Al_2_O_3_ (reference code 98-017-3713, [68]). Nevertheless, it should also be mentioned that *θ*-Al_2_O_3_ has a disordered structure [69,70,71,72]. As such, the peak could also be a result of stacking faults (twinning) or other structural defects in *θ*-Al_2_O_3_ [73,74,75]. The precise peak assignment was not possible, owing to the difficulty to unambiguously distinguish the various Al_2_O_3_ polymorphs by XRD technique alone.

**Figure 8:**
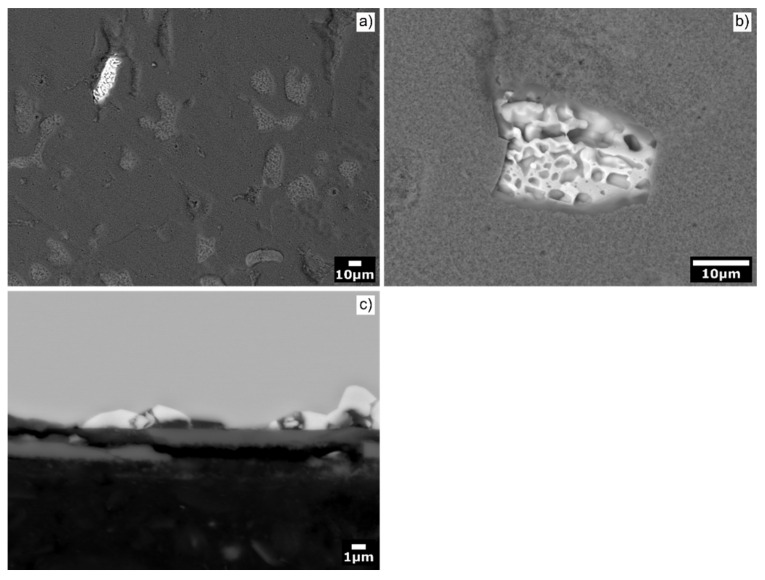
Scale microstructure around *β*-AlCo dendrites of the Al_71_Co_29_ alloy: (**a**) an overview; (**b**) a detailed view; (**c**) cross section image of the scale.

The alumina scale was well adherent to the substrate (Figure 4a and Figure 5a). Nevertheless, locally, a detachment of the scale on the Al_71_Co_29_ alloy was observed (Figure 8). A scale delamination was found preferentially around *β*-AlCo dendrites. The situation is shown in Figure 8c. An explanation of the layer spallation could reside in a mechanical stress developed during oxide growth. The stress is formed due to different molar volumes of the oxide and the underlying original metal substrate [76]. The stress generated in the oxide during excessive growth may lead to crack formation in the scale. The pores, cracks and other defects facilitate the access of molecular oxygen to the metal substrate.

Another interesting feature was the porosity of *β*-AlCo dendrites located beneath the spalled scale of the Al_71_Co_29_ alloy (Figure 8b). The voids in *β*-AlCo were not observed before oxidation (Figure 2). The voids were thus formed during reaction, probably because of rapid aluminum outward diffusion from the metal surface. During oxidation, metallic species diffuse out from the alloy bulk to the alloy/oxide interface. The Al atoms, however, leave behind their vacant sites. The vacancies may have coalesced into larger defects, giving rise to the observed macroscopic porosity. The oxide spallation has not been observed on the Al_76_Co_24_ alloy. The Al_76_Co_24_ alloy is composed of structurally complex intermetallic phases (Z-Al_3_Co, m-Al_13_Co_4_ and Al_9_Co_2_, Figure 3). The surfaces of these phases are typically Al-rich [77]. Aluminum necessary for the scale growth was readily available at the surface of complex intermetallics. As such, the diffusion of Al atoms from these phases was less rapid compared to *β*-AlCo.

The scale of the Al_76_Co_24_ alloy did not show any spallation. It was well adherent to the substrate and had a wave-like morphology (Figure 5). The wave-like morphology of the scale could be indicative of epitaxial growth. The XRD pattern showed a preferential orientation of *θ*-Al_2_O_3_ grains in (002) crystallographic plane (Figure 6). The *θ*-Al_2_O_3_ (400) peak was not observed. The Al_76_Co_24_ alloy was primarily composed of m-Al_13_Co_4_, with small amounts of Z-Al_3_Co and Al_9_Co_2_ (Table 1). A preferred orientation is typical for m-Al_13_Co_4_ phase [26,78]. It is therefore likely that the *θ*-Al_2_O_3_ phase was formed with an orientation relationship to the m-Al_13_Co_4_ phase. This may be reflected in the preferential orientation of the *θ*-Al_2_O_3_ grains, as a result of which the intensities of the peaks of this phase change and some may disappear [79,80].

The oxide layer on the Al_71_Co_29_ alloy, on the other hand, was more uniform (Figure 4). The preferential layer growth is a self-limited process driven by atomic diffusion, and surface energy minimization [81,82]. It requires a certain concentration and certain mobility of Al atoms on the surface. The Al_71_Co_29_ alloy was primarily composed of Al_5_Co_2_. The Al_76_Co_24_ alloy, on the other hand, was mainly composed of m-Al_13_Co_4_ (Table 1). The surface of Al_5_Co_2_ is terminated at specific bulk layers (Al-rich puckered layers) where various fractions of specific sets of Al atoms are missing [83]. The surface of m-Al_13_Co_4_ has a higher density of Al atoms when compared to Al_5_Co_2_. Previous investigations on Al_13_Co_4_ (100) showed that it was terminated by a dense aluminum topmost layer [84]. Therefore, the conditions for the atomic diffusion and subsequent oxide growth on the Al_76_Co_24_ and Al_71_Co_29_ alloys were different. The preferential oxide growth was less favored on the Al_71_Co_29_ alloy.

**Figure 9:**
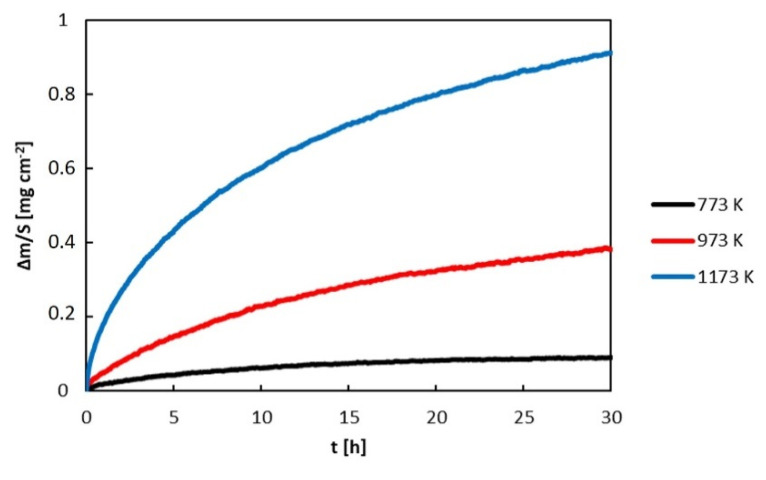
Mass gain of the Al_71_Co_29_ alloy during isothermal oxidation in flowing synthetic air.

**Figure 10:**
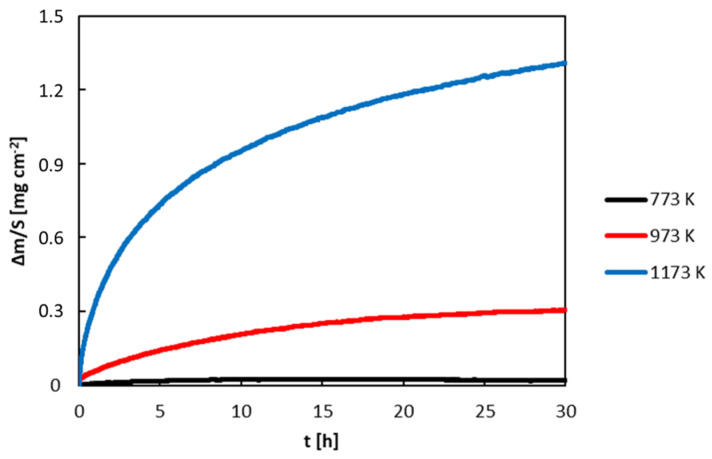
Mass gain of the Al_76_Co_24_ alloy during isothermal oxidation in flowing synthetic air.

The mass gain of the samples was recorded by simultaneous thermogravimetry (TGA). Thermogravimetric curves of the Al_71_Co_29_ and Al_76_Co_24_ alloys are presented in Figure 9 and Figure 10. The mass gain of the samples was increasing with increasing time. The kinetic curves obeyed a parabolic behavior.

The parabolic oxidation could be described by the following equation

(1) $${\left(\frac{\Delta m}{S}\right)}^{2}=\;k_{p}t\;+\;C$$

In this equation, $\frac{\Delta m}{S}$ represents the specific mass gain (mass increase per unit area), k_p_ is the parabolic constant, t is the annealing time and C is the integration constant. The plot of ${\left(\frac{\Delta m}{S}\right)}^{2}$ versus t was linear and the slope of the line represented the parabolic rate constant. The k_p_ values are collected in Table 2. The rate constants of the alloys are found between 1.6 × 10^−14^ and 2.5 × 10^−11^ g^2^ cm^−4^ s^−1^. The obedience of the parabolic behavior shows that the oxidation process of the Al_71_Co_29_ and Al_74_Co_26_ alloys was controlled by ionic diffusion in the scale.

Oxidation is a thermally activated process. The parabolic rate constants increase with increasing temperature. The activation energy of oxidation thus could be obtained from the following equation

(2) $$\log k_{p}\;=\;\log A\;-\;0.434\frac{E_{A}}{\mathrm{RT}}$$

**Figure 11:**
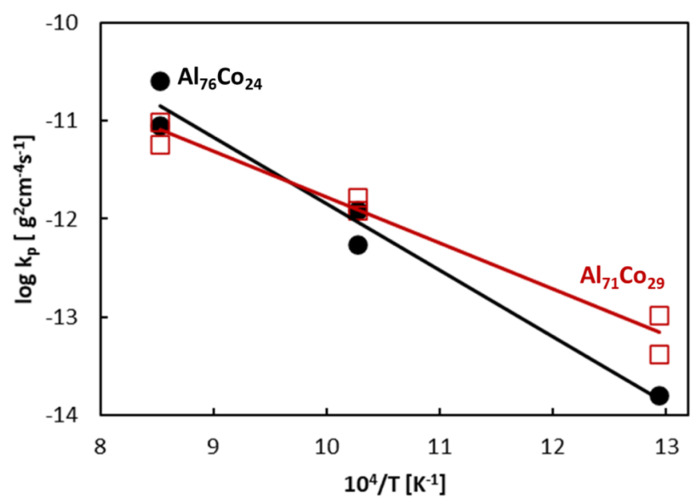
Temperature dependence of parabolic rate constants of the Al-Co alloys.

In this equation, A is a constant, E_A_ is the activation energy, R is the molar gas constant (8.3144 J K^−1^ mol^−1^) and T is the absolute temperature (in K). The plot of rate constants at different temperatures is presented in Figure 11. The rate constants follow the Arrhenius-type behavior. Activation energy has been found from the slope of lines given in Figure 11. The activation energy of the Al_71_Co_29_ alloy is 90 kJ mol^−1^ and the activation energy of the Al_76_Co_24_ alloy is 129 kJ mol^−1^. These activation energies are comparable to activation energies for Al oxidation reported by previous studies [85,86].

Results presented above show that a protective scale has been formed on the surface of complex metallic alloys. The rate constants were relatively low, and a thin alumina scale was found on the surface (Figure 4 and Figure 5). Alumina is formed by the following reaction

(3) $$\frac{4}{3}\mathrm{Al}\;+\;O_{2}\;\to \;\frac{2}{3}{\mathrm{Al}}_{2}O_{3}$$

**Figure 12:**
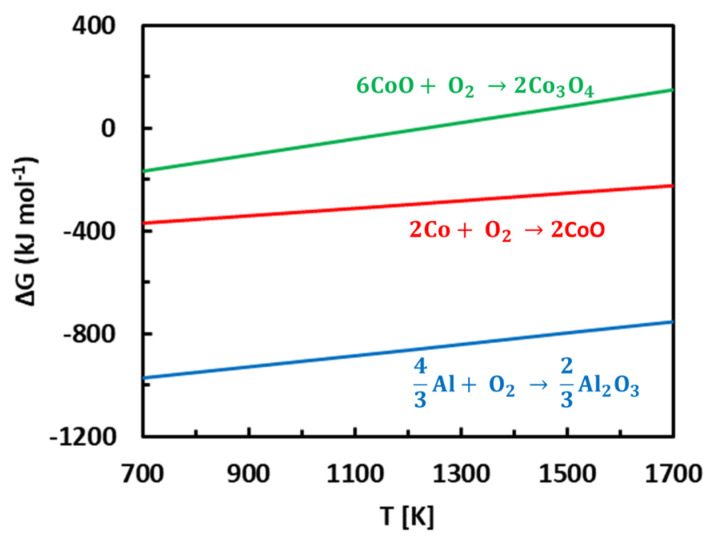
Gibbs free energies of metal oxidation reactions at elevated temperatures, redrawn from [54].

The Gibbs energy (ΔG) of reaction (3) is given in Figure 12. ΔG(Al_2_O_3_) is very low which indicates a strong affinity of aluminum towards oxygen. In principle, cobalt oxidation in the Al-Co alloys is also possible. This reaction can be given by the following equation

(4) $$2\mathrm{Co}\;+\;{\;O}_{2}\;\to \;2\mathrm{CoO}$$

Nevertheless, ΔG reaction (4) is considerably larger compared to Gibbs free energy for aluminum oxidation (Figure 12). A further oxidation of CoO is even more energetically demanding [87,88]. Therefore, CoO tends to decompose in reaction with Al. The reaction can be expressed by the following equation

(5) $$\frac{4}{3}\mathrm{Al}\;+\;\;2\mathrm{CoO}\;\to \;\frac{2}{3}{\mathrm{Al}}_{2}O_{3}\;+\;\mathrm{Co}$$

In this disproportionation reaction, CoO is reduced, and Al oxidized. The Gibbs energy of reaction (5) is negative. Therefore, the selective oxidation of aluminum in the Al-Co alloys is thermodynamically possible.

**Figure 13:**
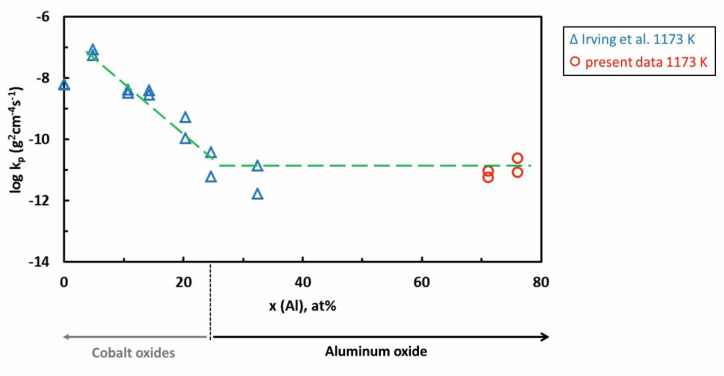
Variation of parabolic rate constants of Al-Co alloys with increasing aluminum atomic fraction.

The oxidation of Co-rich Al-Co alloys was previously studied by Irving et al. [61]. The alloys were studied in the as-cast state. The authors studied several Co-xAl alloys with x = 0–32 at.%. Alloys with small Al concentration (< 10 at.%) formed a single CoO layer. Cobalt oxide layer grew with a considerably higher corrosion rate compared to Al_2_O_3_. At intermediate Al concentrations (10–20 at.%), the authors found that an inner layer of Al_2_O_3_ started to form below the outer CoO scale. With increasing aluminum concentration, a continuous Al_2_O_3_ scale has been developed. The comparison of the present results with those from literature is given in Figure 13. Our data complement the previous results of Irving et al. The continuous Al_2_O_3_ scale forms a barrier to cobalt diffusion. It hinders the nucleation and growth of cobalt oxides. Irving et al. found that a protective alumina scale can be formed when Al concentration 24 at.% at 1173 K is reached. Comparable minimum Al concentrations required to form the external alumina scale were also found for the Ni-Al and Fe-Al alloys [89].

**Figure 14:**
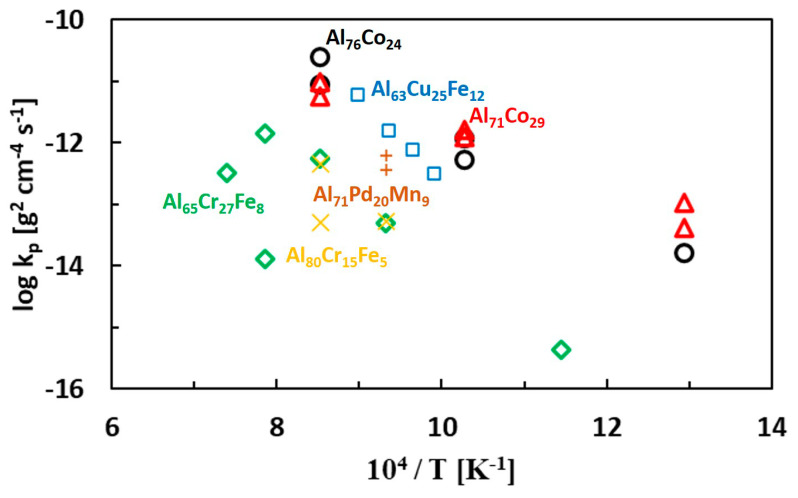
Parabolic rate constants for metal oxidation of Al-TM complex metallic alloys.

The comparison of the present results with previously studied complex metallic alloys is provided in Figure 14. Kinetics of oxidation of Al-Cu-Fe and Al-Pd-Mn quasicrystal surfaces was studied in synthetic air [66,90]. High temperature oxidation kinetics of Al-Cr-Fe complex metallic alloys was studied in pure oxygen [91]. Our data are comparable to Al-Cu-Fe and Al-Pd-Mn alloys. The parabolic rate constants of the Al-Cr-Fe complex metallic alloys are lower compared to the remainder of the alloys. The oxidation resistance of the Al-Fe-TM (TM = Cr, Cu) alloys is related to the chemical composition of the oxide scale. The scale found in Al-Cu-Fe alloys was alumina. The scale formed in Al-Cr-Fe complex metallic alloys, however, was composed of Al_2_O_3_ and (Al_0.9_Cr_0.1_)_2_O_3_. The second scale component provided an additional barrier against corrosion. Chromium as a third alloying element may improve the overall oxidation resistance of the alloy. The corrosion resistance of alumina forming alloys alloyed with chromium is higher compared to alloys without Cr. When a sufficient Cr concentration is available, a complete chromia scale can be formed on top of the alumina scale [92,93]. The duplex Al_2_O_3_/Cr_2_O_3_ scale has an outstanding corrosion resistance.

Previous authors also studied the microstructure evolution of the oxide scale. In early oxidation stages, *γ*-Al_2_O_3_ on the Al_63_Cu_25_Fe_12_ alloy was formed with an orientational relationship to the underlying Al-Cu-Fe quasicrystal [66]. *γ*-Al_2_O_3_ continued to grow as *θ*-Al_2_O_3_ until the oxide layer of several hundred nanometers has been formed. *θ*-Al_2_O_3_ was later transformed into the thermodynamically stable *α*-Al_2_O_3_. *α*-Al_2_O_3_ continued to grow with a nodular morphology. The oxide-nodule formation changed the growth mechanism. A protective layer formation was no longer observed. A massive spallation occurred after few days of oxidation [66]. A spallation of Al_2_O_3_ scale from the oxidized Al-Cr-Fe surfaces at high temperatures was also observed [91]. The massive oxide spallation has not been observed in the present study. However, the oxide spallation was observed locally on *β*-AlCo dendrites (Figure 8). It is possible that further stresses in the scale could develop during long term annealing. Therefore, further experiments on the Al-Co complex metallic alloys are required to study the effects of long-term annealing and/or thermal cycling on the oxidation behavior. These studies could shed further light into the long-term oxidation resistance for practical applications of the Al-Co alloys at high temperatures.

## 4. Conclusions

In the present work, the oxidation behavior of the Al_71_Co_29_ and Al_76_Co_24_ alloys (concentration in at.%) was studied at 773–1173 K in air. The alloys were prepared by rapid solidification of Al and Co lumps in argon. The alloys were studied in as-solidified state. The following conclusions can be drawn:

1. The alloys were composed of different microstructure constituents. The Al_76_Co_24_ alloy was composed of Al_9_Co_2_, m-Al_13_Co_4_ and Z-Al_3_Co. The Al_71_Co_29_ alloy consisted of Z-Al_3_Co, Al_5_Co_2_ and *β*-AlCo. The precipitation sequences of the constituents were explained based on the equilibrium Al-Co phase diagram and rapid solidification processes taking place during casting.
2. During oxidation in air, aluminum in the alloys was selectively oxidized and a protective alumina scale was formed on the alloy surfaces. The oxidation kinetics followed a parabolic rate law. The rate constants of the alloys were between 1.63 × 10^−14^ and 8.83 × 10^−12^ g cm^−4^ s^−1^, depending on the annealing temperature. The activation energies of oxidation were 90 kJ mol^−1^ for the Al_71_Co_29_ alloy and 123 kJ mol^−1^ for the Al_76_Co_24_ alloy, respectively.
3. The scale of the Al_76_Co_24_ alloy was adherent to the substrate and had a wave-like morphology. At 1173 K, a preferential orientation of *θ*-Al_2_O_3_ in (002) crystallographic plane was found. The scale on the Al_71_Co_29_ alloy was more uniform and spallation was observed locally on the dendritic *β*-AlCo.
4. The oxidation kinetics of the Al_71_Co_29_ and Al_76_Co_24_ alloys was comparable to previously studied Al_24_Co_76_ and Al_32_Co_68_ alloys where a continuous alumina scale had been formed. The increased Al concentration contributes to the alloy’s corrosion resistance. The continuous Al_2_O_3_ scale forms a barrier to cobalt diffusion, and it hinders the nucleation and growth of cobalt oxides.

## Figures and Tables

**Table 1:** Chemical compositions of microstructure constituents observed in the as-solidified Al_71_Co_29_ and Al_76_Co_24_ alloys.

**Al_71_Co_29_ Alloy**				
**Microstructure Constituent**	**Phase Identified [57]**	**Al [at.%]**	**Co [at.%]**	**Volume Fraction [%]**
Light-grey	*β*-AlCo	55.2 ± 0.9	44.8 ± 0.9	8
Medium-grey	Al_5_Co_2_	71.9 ± 0.4	28.1 ± 0.4	86
Dark-grey	Z-Al_3_Co	74.6 ± 0.4	25.4 ± 0.4	6
**Al_76_Co_24_ Alloy**				
**Microstructure Constituent**	**Phase Identified [58]**	**Al [at.%]**	**Co [at.%]**	**Volume Fraction [%]**
Light-grey	Z-Al_3_Co	74.4 ± 0.1	25.6 ± 0.1	5
Medium-grey	m-Al_13_Co_4_	75.2 ± 0.2	24.8 ± 0.2	83
Dark-grey	Al_9_Co_2_	81.5 ± 0.1	18.5 ± 0.1	12

**Table 2:** Parabolic rate constants of the Al_71_Co_29_ and Al_76_Co_24_ alloys in air.

	**k_p_ [g^2^ cm^−4^ s^−1^]**	
**Al_71_Co_29_ Alloy**		
**773 K**	**973 K**	**1173 K**
1.04 × 10^−13^ (0–15 h)	1.64 × 10^−12^ (0–15 h)	9.71 × 10^−12^ (0–15 h)
4.20 × 10^−14^ (20–30 h)	1.23 × 10^−12^ (15–30 h)	5.81 × 10^−12^ (15–30 h)
**Al_76_Co_24_ Alloy**		
**773 K**	**973 K**	**1173 K**
1.63 × 10^−14^	1.21 × 10^−12^ (0–15 h)	2.54 × 10^−11^ (0–10 h)
	5.43 × 10^−13^ (15–30 h)	8.83 × 10^−12^ (20–30 h)
